# Reinforcement learning accounts for moody conditional cooperation behavior: experimental results

**DOI:** 10.1038/srep39275

**Published:** 2017-01-10

**Authors:** Yutaka Horita, Masanori Takezawa, Keigo Inukai, Toshimasa Kita, Naoki Masuda

**Affiliations:** 1 National Institute of Informatics, 2-1-2 Hitotsubashi, Chiyoda-ku, Tokyo 101-8430, Japan; 2 JST, ERATO, Kawarabayashi large graph project, c/o Global Research Center for Big Data Mathematics, NII, 2-1-2 Hitotsubashi, Chiyoda-ku, Tokyo 101-8430, Japan; 3 Department of Behavioral Science, Hokkaido University, N10W7, Kita-ku, Sapporo, 060-0810, Japan; 4 Center for Experimental Research in Social Sciences, Hokkaido University, N10W7, Kita-ku, Sapporo, 060-0810, Japan; 5 Institute of Social and Economic Research, Osaka University, 6-1, Mihogaoka, Ibaraki, Osaka, 567-0047, Japan; 6 Department of Engineering Mathematics, University of Bristol, Merchant Venturers Building, Woodland Road, Clifton, Bristol BS8 1UB, United Kingdom

## Abstract

In social dilemma games, human participants often show conditional cooperation (CC) behavior or its variant called moody conditional cooperation (MCC), with which they basically tend to cooperate when many other peers have previously cooperated. Recent computational studies showed that CC and MCC behavioral patterns could be explained by reinforcement learning. In the present study, we use a repeated multiplayer prisoner’s dilemma game and the repeated public goods game played by human participants to examine whether MCC is observed across different types of game and the possibility that reinforcement learning explains observed behavior. We observed MCC behavior in both games, but the MCC that we observed was different from that observed in the past experiments. In the present study, whether or not a focal participant cooperated previously affected the overall level of cooperation, instead of changing the tendency of cooperation in response to cooperation of other participants in the previous time step. We found that, across different conditions, reinforcement learning models were approximately as accurate as a MCC model in describing the experimental results. Consistent with the previous computational studies, the present results suggest that reinforcement learning may be a major proximate mechanism governing MCC behavior.

Cooperation is everywhere in human society^1^,^2^,^3^. In many instances of cooperation, social dilemma exists such that it is apparently more rational for an individual not to cooperate, whereas the individual is better off if everybody cooperates. Using social dilemma games including the prisoner’s dilemma game (PDG) and public goods game (PGG), various mechanisms governing cooperative human and animal behavior in social dilemma situations have been studied, in both theory and experiments.

One core mechanism that facilitates cooperation in social dilemma situations is direct reciprocity^4^,^5^. When dyadic interactions between the same individuals are repeated, each of them is motivated to cooperate because doing so increases future payoffs in general. There is ample evidence that humans show direct reciprocity behavior^6^,^7^,^8^. Similar behavior has been observed beyond dyadic relationships of individuals. In the PGG, where individuals interact in a group, a substantial fraction of people is known to implement conditional cooperation (CC). With CC, cooperation increases if others in the same group have cooperated a lot^9^,^10^,^11^,^12^,^13^,^14^,^15^,^16^,^17^,^18^ (also see ref. 19 for CC behavior in a multiplayer PDG; also see refs 20, 21, 22, 23, 24, 25, 26, 27, 28 for theoretical/computational studies of CC). In a related vein, cooperation and/or defection in the PGG can be contagious^29^,^30^. Furthermore, in multiplayer PDGs, moody conditional cooperation (MCC) behavior rather than CC has been observed^31^,^32^,^33^,^34^,^35^. By definition, MCC dictates that an individual tends to cooperate if many others have cooperated (i.e., CC) and the focal individual has cooperated the last time. If an individual has defected the last time, the same individual would not increase cooperation even if many others have cooperated the last time^31^,^32^,^33^,^34^,^35^. See refs 32, 33, 34 and 36, 37, 38 for theoretical/computational studies of MCC.

Another stream of approach to explain cooperation in repeated games is to assume reinforcement learning. In short, individuals obeying reinforcement learning would enhance the currently taken action if it has turned to be rewarding and vice versa. Different models of reinforcement learning have been fit for describing human cooperation^39^,^40^,^41^,^42^,^43^. Computational models of reinforcement learning for social dilemma games have been studied even longer^6^,^44^,^45^,^46^.

In our recent computational study, we showed that a reinforcement learning model could explain CC and MCC patterns without explicitly assuming a CC or MCC behavioral rule^38^. In the present study, we asked the same question in behavioral experiments. Using two types of multiplayer social dilemma games, we compared explanatory powers of multiplayer counterparts of direct reciprocity (i.e., CC and MCC) and two models of reinforcement learning. We fitted these models to our data obtained from behavioral experiments. Our participants played both a dyadic PDG^19^,^31^,^32^,^33^,^34^,^35^, in which MCC had been previously observed, and the PGG^9^,^10^,^11^,^12^,^13^,^14^,^15^,^16^,^17^,^18^,^43^, in which CC or reinforcement learning had often been considered.

## Results

### Experimental setup

Each participant played a repeated multiplayer PDG (referred to as PDG for short in the following) and the repeated PGG. In each type of game, the participants formed a four-person group and submitted their decisions over rounds. In each round of the PDG, each participant received an endowment and selected either cooperation (C) or defection (D). A participant that selected C donated 3*c*, and *c* is transferred to each of the three other participants in the group. Each of the three participants then received a benefit of *b* (= 2*c*). A participant that selected D kept the endowment. In this case, the other three participants in the group received nothing. The participants were not allowed to simultaneously select C toward some participants and D toward the others. In each round of the PGG, each participant received an endowment and decided on the amount of contribution, *x* (= 0, 1, …, 25), to a common pool. The sum of the contributions by the four group members were multiplied by 1.6 and then imparted equally to the four participants.

The participants played 20 rounds (i.e., they submitted decisions 20 times each) in each type of game. In every round except the first round, each participant was informed of the decisions and payoffs that he/she and the three current group members had received in the previous round.

Each participant played the two types of game in either the so-called fixed or mixed conditions, but not both. In the fixed treatment, participants played the games with same three partners in all rounds. In the mixed treatment, the three partners were randomly shuffled in every round. See Methods for details. In total, we retrieved 4,000 decisions and used them as the unit of analysis.

### Fraction of cooperation

**Figure 1:**
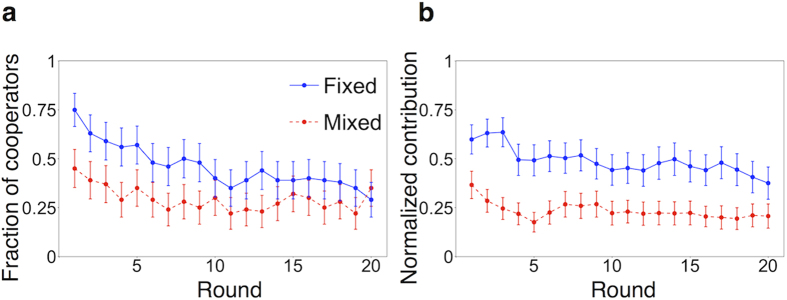
Time courses of the amount of cooperation. (**a**) Fraction of cooperating participants in the PDG. (**b**) Mean normalized contribution in the PGG. The solid and dashed lines correspond to the fixed and mixed treatments, respectively. The error bars represent 95% confidence intervals (±1.96 × *SE*).

The fraction of cooperators averaged over all participants in the PDG and the mean fraction of contribution in the PGG are plotted against the round in Fig. 1a,b, respectively. In the PGG, if a participant contributed 10 monetary units, for example, out of the total endowment, which was equal to 25, the fraction of contribution was defined to be 10/25 = 0.4. In both types of game, cooperation declined over rounds regardless of the interaction type (i.e., fixed or mixed). With the overall level of cooperation included, this result is consistent with the previous results using the PDG^19^,^31^,^32^,^33^,^34^,^35^,^47^ and the PGG^48^,^49^,^50^.

Figure 1 indicates that the fraction of cooperation is consistently larger in the fixed than mixed treatment. This is probably because the participants would count on direct reciprocity, i.e., cooperation owing to repeated interactions with the same partners^4^,^5^, in the fixed but not in the mixed treatment. For the PGG, this result is consistent with the previous results^9^,^48^. For the PDG, this result is inconsistent with previous literature reporting similar fractions of cooperators between the fixed and mixed treatments^19^,^47^. The reason for this discrepancy is unclear. It may be due to the different experimental settings such as payoff values, network structure, and the size of the group. Although cooperation was more frequent in the fixed than mixed conditions in other studies^31^,^33^, consistent with the present results, all participants in these experiments were exposed to the fixed treatment first and then to the mixed treatment.

Across participants, the fraction of cooperation in the PDG averaged over the rounds was positively correlated with the amount of contribution in the PGG averaged over the rounds. This was the case in both fixed and mixed treatments (fixed: *r *= 0.28, *p *< 01; mixed: *r *= 0.58, *p *< 01; see Supplementary Fig. S2 for a scatter plot).

### Conditional cooperation and moody conditional cooperation

**Figure 2:**
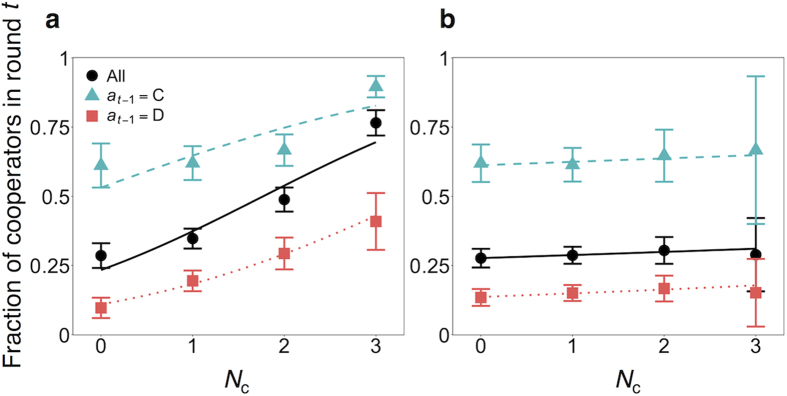
Fraction of cooperation in the PDG as a function of the number of the other group members that cooperated in the previous round, *N*_*c*_. (**a**) Fixed treatment. (**b**) Mixed treatment. The circles represent the fraction of cooperators not conditioned on the action of the focal participant in round *t*−1, *a*_*t*−1_. The triangles and squares represent the fraction of cooperators conditioned on *a*_*t*−1_ = C and *a*_*t*−1_ = D, respectively. The error bars represent the 95% confidence intervals. The solid curves represent fitting of the CC model. The dashed and dotted curves represent the probability of C conditioned on *a*_*t*−1_ = C and *a*_*t*−1_ = D, respectively, fitted by the MCC model.

We measured the probability of cooperation in the PDG as a function of the number of cooperative other group members in the previous round, denoted by *N*_c_ ( = 0, 1, 2, or 3), and the action of the focal participant in the previous round, denoted by *a*_*t*−1_ (Table 1). The results for the fixed and mixed treatments, aggregated over the participants and rounds, are shown in Fig. 2a,b, respectively.

In the fixed treatment, cooperation increased with *N*_c_ when not conditioned on *a*_*t*−1_, implying conditional cooperation (CC), as indicated by the circles in Fig. 2a. This was also the case when the probability of cooperation was conditioned on *a*_*t*−1_ (triangles and squares). A participant tended to cooperate more when he/she had cooperated in the previous round, consistent with moody conditional cooperation (MCC) behavior found in the previous experiments^31^,^32^,^33^,^34^,^35^. However, the pattern of MCC is different between the present and previous results. In the previous experiments^31^,^32^,^33^,^34^,^35^, cooperation increased as *N*_c_ increased when *a*_*t*−1_ = C, whereas cooperation decreased as *N*_c_ increased or was almost independent of *N*_c_ when *a*_*t*−1_ = D. In the present experiments, cooperation increased as *N*_*c*_ increased regardless of *a*_*t*−1_, and *a*_*t*−1_ affected the baseline level of cooperation (triangles and squares in Fig. 2a). In the mixed treatment, CC or MCC was absent (Fig. 2b), consistent with a previous experimental study^31^. This is probably because direct reciprocity is absent in the mixed treatment^31^.

**Figure 3:**
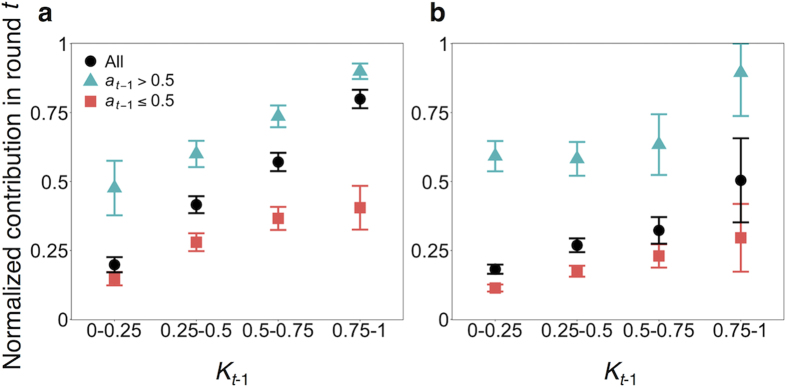
Contribution in the PGG as a function of the average normalized contribution by the other group members in the previous round, *K*_*t*−1_. (**a**) Fixed treatment. (**b**) Mixed treatment. Due to the continuous nature of *K*_*t*−1_, we categorized the decisions into four bands according to the value of *K*_*t*−1_ and then calculated the statistics for the data in each band. Error bars represent the 95% confidential intervals. The circles represent the participant’s normalized contribution, *a*_*t*_, not conditioned on the same participant’s contribution in the previous round, *a*_*t*−1_. The triangles and squares represent *a*_*t*_ of high (*a*_*t*−1_ > 0.5) and low (*a*_*t*−1_ ≤ 0.5) contributors in the previous round, respectively.

The corresponding results for the PGG are shown in Fig. 3. In the figure, the normalized contribution per player, denoted by *a*_*t*_, averaged over all participants and rounds, is plotted against the average fraction of contribution by the other group members in the previous round, denoted by *K*_*t*−1_ (0 ≤ *K*_*t*−1_ ≤ 1; Table 1). The contribution increased as *K*_*t*−1_ increased (circles), consistent with CC patterns found in the previous experiments using the PGG^10^,^17^. This result held true in both fixed and mixed treatments (fixed: *r* = 0.55, *p *< 0.01; mixed: *r *= 0.21, *p *< 0.01). Also shown in Fig. 3 is *a*_*t*_ conditioned on the fraction of contribution by the same player in the previous round, *a*_*t*−1_, as well as on *K*_*t*−1_. The results for high contributors (*a*_*t*−1_ > 0.5) and low contributors (*a*_*t*−1_ ≤ 0.5) in the previous round are shown by the triangles and squares, respectively. The high contributors in round *t *− 1 contributed more in round *t* than the low contributors in round *t *− 1 did in both fixed and mixed treatments. In the fixed treatment, for both high and low contributors, *a*_*t*_ was positively correlated with *K*_*t*−1_ (high contributors, *r *= 0.42, *p *< 0.01; low contributors, *r *= 0.33, *p  *< 0.01). This was also the case in the mixed treatment (high contributors: *r *= 0.12, *p *< 0.05; low contributors: *r *= 0.21, *p *< 0.01). In sum, MCC similar to that observed in the PDG was present in both treatments in the PGG.

### Model fitting

We fitted the following four models to the behavioral data. All models were intended to explain the level of cooperative behavior in round *t* (0 ≤ *p*_*t*_ ≤ 1, Table 1) given the information up to the previous round. For the PDG, the dependent variable was the probability of selecting C in round *t*. For the PGG, the dependent variable was the normalized contribution in round *t*. The precise definition of the models is given in Methods.

The first model intends to capture CC behavior. By definition, in the CC model, a focal player cooperates much if the other participants have cooperated much in the previous round. In the second model, i.e., the MCC model, the level of cooperation depends on the amount of cooperation made by the others in the previous round and additionally on *a*_*t*−1_. The third model is the Bush-Mosteller (BM) model of reinforcement learning. We used a variant of the BM model with a fixed reference (also called aspiration) level^38^,^46^ (see ref. 45 for a similar model). In the BM model, we reinforce the current action by revising *p*_*t*_ if a player is satisfied with the outcome of the game. Otherwise, the current action will be anti-reinforced. The level of the satisfaction is measured by the difference between the obtained earning and the fixed reference level denoted by *A*. The fourth model is the Roth-Erev (RE) model of reinforcement learning, in which the attraction score of all options was updated after each round^39^,^40^,^41^,^51^. By definition, a player tends to select an action with a high attraction score.

### Model selection

We fitted each model to the experimental data using maximum likelihood estimation. We evaluated the goodness-of-fit of each model using the Akaike information criterion (AIC) and the mean squared error (MSE) (see Methods).

For the PDG, the inferred parameter values are shown in Supplementary Table S1 for the four models. We separately used the data in the fixed treatment and those in the mixed treatment to carry out maximum likelihood estimation. For the CC and MCC models, the fixed treatment yielded much larger estimate of the impact of *K*_*t*−1_ on *p*_*t*_ (parameter *α*_1_ in equations (3) and (4)) than the mixed treatment did. This result is consistent with those shown in Fig. 2, i.e., CC behavior is eminent in the fixed but not mixed treatment. These models predicted *p*_*t*_ values (lines in Fig. 2) reasonably close to the experimental data (symbols in Fig. 2). For the MCC model, the influence of the previous action (i.e., *a*_*t*−1_) on the present action (i.e., *a*_*t*_) was significant in both treatments (parameter *α*_3_ in equation (4) being significantly positive). However, the interaction term (*α*_4_*a*_*t*−1_*K*_*t*−1_ in equation (4)) was not significant in either treatment. Therefore, MCC behavior in the sense that the sensitivity of *p*_*t*_ to *K*_*t*−1_ depends on whether *a*_*t*−1_ = 1 (i.e., C) or *a*_*t*−1_ = 0 (i.e., D)^31^,^32^,^33^,^34^,^35^ was absent. For the BM model, the inferred reference level (*A* in equation (5)) was lower than the smallest possible payoff value (i.e., 10 yen). Therefore, according to the model, the player was satisfied by any outcome, and only the amount of reinforcement, but not the direction of reinforcement, depended on the payoff. This pattern of learning is similar to that of the RE model by construction. For the RE model, the inferred value of *λ* was positive in both the fixed and mixed treatments. Therefore, the choice of the players depended on the payoff in the previous round and did not occur randomly.

The log likelihood, AIC, and MSE for the different models are shown in Table 2. A smaller AIC or MSE value implies a better fit of a model to the data. The AIC value for the RE model was the smallest among the four models in the fixed treatment, whereas that for the BM model was the smallest in the mixed treatment. The AIC values for the reinforcement learning models (i.e., BM and RE models) were smaller than the AIC value for the MCC model, which was smaller than the AIC value for the CC model, in both treatments. The MSE values were statistically similar among the MCC, BM, and RE models and smaller than for the CC model in the fixed treatment. In the mixed treatment, the MSE values for the BM and RE models were statistically smaller than those for the MCC and CC models (Table 2). Therefore, we conclude that the two reinforcement learning models (i.e., BM and RE) explain the behavioral data better than the CC model does. Our MCC model was roughly as accurate as the reinforcement learning models, but the reinforcement learning models explained behavioral data better than the CC and MCC models did, especially when CC behavior was absent, i.e., in the mixed treatment. We confirmed that numerical simulations of the BM and RE models produced the relationship between the probability of cooperation (i.e., *p*_*t*_) and the number of cooperating others in the previous round (i.e., *N*_c_), which was consistent with MCC patterns observed in our experiments (Supplementary Fig. S3).

For the PGG, the inferred parameter values are shown in Supplementary Table S2. Whereas the sensitivity of *p*_*t*_ to *K*_*t*−1_ (i.e., *α*_1_) for the CC and MCC models in the PDG was large only in the fixed treatment, the same sensitivity in the PGG was large in both of the fixed and mixed treatments. This result implies that CC behavior was present in the PGG regardless of the treatment. For the MCC model, the effect of *a*_*t*−1_ on *a*_*t*_ (i.e., *α*_3_ in equation (4)) was significantly positive in both treatments. These results are consistent with those shown in Fig. 3, where both low and high contributors in the previous round increased their contribution if other group members had increased the contribution. We found a significant interaction effect between *a*_*t*−1_ and *K*_*t*−1_ (i.e., *α*_4_ in equation (4) was significantly larger than zero) in the fixed but not in the mixed treatment. Therefore, in the fixed treatment, both high and low contributors increased their cooperation depending on the level of cooperation by the other three members, while high contributors did so more than low contributors did. Similar to the case of the PDG, the values of *A* for the BM model were lower than the smallest possible payoff value in both the fixed and mixed treatments. The results for the RE model in the PGG were qualitatively similar to those in the PDG as well.

Table 2 indicates that, in both treatments in the PGG, the AIC value for the CC model was the largest (i.e., worst) among the four models. In the fixed treatment, our MCC model provided the best fit to the data in terms of the AIC. In the mixed treatment, the two reinforcement learning models (i.e., BM and RE) were better than the MCC model in terms of the AIC. Under both treatments, the MSE values for the MCC, BM, and RE models were similar and smaller than those for the CC model. Therefore, we conclude that, in the PGG game, the MCC model and the two reinforcement learning models have similar explanatory power, and all of them outperform the CC model. We confirmed that numerical simulations of the BM and RE models produced the relationship between the normalized contribution in the present round and the average contribution by the other group members in the previous round (i.e., *K*_*t*−1_) consistent with MCC patterns (Supplementary Fig. S4).

Directional learning is another variant of reinforcement learning, often applied to the PGG^43^. We fitted a directional learning model to our PGG data. By definition of directional learning, if an increased contribution in the previous round has yielded a large reward, then a player would contribute more in the next round, and vice versa (see Supplementary Method for the definition). The AIC value for the directional learning model was larger than that for the CC, MCC, BM, and RE models in both the fixed and mixed treatments. The MSE value for the directional learning model was also larger than that for the other four models in both treatments (Supplementary Table S3 and Fig. S4). Therefore, the directional learning model does not account for our experimental data.

## Discussion

We analyzed behavioral patterns of human participants engaged in two social dilemma games, i.e., the multiplayer PDG and the PGG. Phenomenologically, we found MCC patterns in both types of game. To the best of our knowledge, the present study shows MCC patterns in the PGG for the first time. However, there is a major difference between the present MCC patterns and those observed in the previous experiments. In the previous studies, cooperation decreased (or did not vary) after other group members cooperated a lot, if the focal player did not cooperate in the previous round^31^,^32^,^33^,^34^,^35^. In our experiments, cooperation increased with the amount of others’ cooperation in this case, and the focal player’s action in the previous round affected the overall level of cooperation of the same player in the current round. Then, for our behavioral data, we compared the explanatory power of a model of CC, a model of MCC, and two reinforcement learning models in terms of the AIC and MSE values. By maximum likelihood estimation of model fitting, we found that the two reinforcement learning models account for the observed human behavior roughly as accurately as the MCC model did. This result is consistent with our previous computational study that has shown that reinforcement learning may be a proximate mechanism underlying human MCC (and CC) behavior^38^.

MCC patterns have been observed in the PDG^31^,^32^,^33^,^34^,^35^. In our experiments, we observed a type of MCC patterns in the PGG. MCC may be also prevalent in other situations and games. Our results suggest a possibility that reinforcement learning is a common proximate mechanism that explains MCC behavior observed in both the PDG and PGG. Exploring MCC-like behavioral patterns in other types of games and further explaining discovered patterns using reinforcement learning may be fruitful.

Among reinforcement learning models, the BM and RE models better fitted to our data than the directional learning model. This result is consistent with our previous computational study^38^. In contrast, directional learning accounted for behavior of humans involved in the PGG in a previous study, in which the authors compared the accuracy of different behavioral rules using regression analysis^43^. They tested whether or not an explanatory variable encoding a directional learning rule significantly increased cooperation, in parallel with whether other explanatory variables encoding different behavioral rules did so. In contrast, we employed a standard model fitting procedure (i.e., likelihood maximization and comparison of the AIC and MSE values) to compare the different candidates of models to reach our conclusions. The difference in the results between the two studies may owe to a large extent to the difference in the methods to assess performances of the models rather than to the different experimental procedures employed in the two studies.

## Methods

### Ethics statements

The present research was approved by the ethic committee of the National Institute of Informatics, Japan and the Center for Experimental Research in Social Sciences at Hokkaido University, Japan. All participants read and signed informed consent forms before participating. The experiments were carried out in accordance with the approved guideline.

### Participants

A total of 200 undergraduate students (77 females and 123 males; mean age 18.85 [*SD* = 0.91]) at Hokkaido University in Japan participated in the experiment. They were recruited via e-mail from a participant pool.

### Games

We divided the population of the 200 participants into ten subpopulations of 20 participants. Each subpopulation played 20 rounds of either type of game (i.e., PDG or PGG) and then 20 rounds of the other type of game. Before the experiment, participants were not informed of the number of rounds in total (i.e., 20). The order of games was counterbalanced across the subpopulations. In the beginning of each round, 20 participants were divided into five groups of four players each. The participants simultaneously submitted their decisions, which defined a round.

In a round in the PDG, all participants simultaneously played the pairwise gift-giving game (also called the donation game) with each of the three other members in the group. In the beginning of a round, each participant received *y* as an endowment and decided whether to give *c* to each group member (i.e, C) or not (i.e., D). When the participant cooperated, *c* went to each of the three peers in the group, who received *b* (=2*c*). Then, the focal participant lost 3*c*. When the focal participant did not donate, the participant did not pay out anything, and the other members did not gain anything. The participant had to select the same action (i.e., C or D) toward the three peers in the group. The total payoff to a player in round *t*, denoted by *r*(PDG)_*t*_, was given by


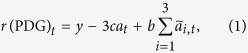


where *a*_*t*_ represents the participant’s action in round *t* (C = 1, D = 0), and 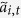 (1 ≤ *i* ≤ 3) is the action of the *i*th other member in the group in round *t*.

In each round of the PGG, the participant contributed *x* out of the endowment *y* (0 ≤ *x *≤ *y*). The normalized contribution in round *t* is defined by *a*_*t*_ =  *x*/*y*. The sum of the contributions from the four group members was multiplied by *m* (>1) and equally divided and imparted to all members, regardless of the amount that the participants contributed. The payoff to a participant in round *t*, denoted by *r*(PGG)_*t*_, was equal to


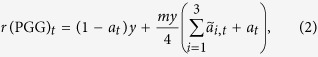


where 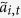 (0 ≤ 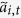 ≤ 1) represents the normalized contribution by the *i*th other member in the group (1 ≤ *i *≤ 3).

We set *y* to 25 Japanese yen (about 0.2 US dollars; 1 yen ≈ 0.008 US dollars) in both the PDG and PGG. We set *b *= 10 yen, *c* = 5 yen, and *m* = 1.6 to make the payoff value the same between the PDG and PGG when all players maximally contributed and when they minimally contributed (see Supplementary Methods for details). In the former case, all four players selected C in the PDG or contributed all the endowment in the PGG, yielding *r*(PDG)_*t*_ = *r*(PGG)_*t*_ = 40 yen. In the latter case, all four players selected D in the PDG or contributed none in the PGG, yielding *r*(PDG)_*t*_ = *r*(PGG)_*t*_ = 0 yen.

### Interaction type

Out of the 200 participants, half of them (i.e., five subpopulations of 20 participants) were assigned to the fixed treatment and the other half to the mixed treatment. Under each treatment, the participants played a repeated PDG composed of 20 rounds and a repeated PGG composed of 20 rounds. In the fixed treatment, four-person groups were formed in a first round of each type of game, and the grouping remained the same until the last round. In the mixed treatment, the group members were randomly reshuffled after each round.

To secure anonymity, each participant was identified by a pseudonym, which was a randomly generated three-letter name. In the beginning of each type of game (i.e., PDG or PGG), a unique pseudonym was issued to each player and displayed on the computer screens of all group members (Supplementary Fig. S1). In the fixed treatment, the four pseudonyms in each group remained the same in all rounds. In the mixed treatment, the pseudonyms were generated randomly in each round. The same pseudonym was never reused.

In every round *t* except the first round (1* *< *t *≤ 20), each participant was informed of the last decisions and earnings (i.e., in round *t *− 1) of everybody in the current group. The information kept displayed on the computer screens when the participants were making a decision in round *t* (Supplementary Fig. S1).

### Procedures

Upon arrival, participants were escorted into a laboratory. In the laboratory, there were 20 tablet computers on desks. Each participant sat in front of a computer. Removable partitions were placed between adjacent participants to prevent them from seeing each other’ face and tablet computer. After all participants had sat, the experimenter gave instruction sheets explaining the rule of either PGG or PDG to the participants and read them aloud. After the instructions finished, the participants answered a questionnaire asking the payoff structure of the game. Those answering incorrectly were led to the correct answers by the experimenter, who mentioned the corresponding part of the written instructions to the participants. After all participants correctly answered the questions, the game started. After finishing 20 rounds in the first type of game, participants were instructed about the rules of the other type of game. Participants were not informed that they would play the opposite type of game until all rounds of the first type of game had finished. After finishing the second type of games, the participants were individually paid according to the earnings summed over all rounds and the two types of games. A participant received 1233.85 yen (approximately 9.87 US dollars) on average.

Participants interacted with other participants using tablet computers connected via a Wi-Fi network. The experimental software was developed by z-Tree^53^.

### CC model

We modeled CC by


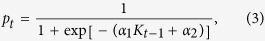


where *α*_1_ and *α*_2_ are parameters controlling the impact of *K*_*t*−1_ on *p*_*t*_ and the baseline level of cooperation when nobody has cooperated in the previous round, respectively. For the PDG, *K*_*t*−1_ was defined as the fraction of the other group members that had selected C in round *t *− 1, i.e., *N*_c_/3.

### MCC model

In MCC, the propensity of cooperation depends on both *K*_*t*−1_ and *a*_*t*−1_. To guarantee 0 ≤ *p*_*t*_ ≤ 1, we modified a previous model for MCC^32^ as follows:


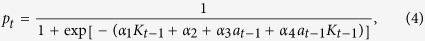


where, for the PDG, *a*_*t*−1_ = 1 and *a*_*t*−1_ = 0 when the focal participant cooperated and defected in the last round, respectively. Among the four parameters *α*_1_, α_2_, α_3_, and α_4_, parameters α_3_ and α_4_ encode MCC behavior. In the previous definition of MCC^32^,^34^, *p*_*t*_ increases as *K*_*t*−1_ increases when *a*_*t*−1_ = 1 and *p*_*t*_ is independent of *K*_*t*−1_ when *a*_*t*−1_ = 0. Here, by not restricting the range of α _1_, α _2_, α _3_, and α _4,_ we allowed *p*_*t*_ to increase as *K*_*t*−1_ increased even when *a*_*t*−1_ = 0, which was consistent with our experimental results.

### BM model

In the BM model of reinforcement learning, the stimulus, *s*_*t*_, which represents the degree of satisfaction perceived by a participant in round *t *− 1, is defined by


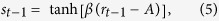


where *r*_*t*−1_ is the earning of the participant in round *t *− 1, and *A* is a reference level. If *r*_*t*−1_ > *A*, the participant is satisfied (*s*_*t*−1_ > 0). If *r*_*t*−1_* *< *A*, the participant is dissatisfied (*s*_*t*−1_* *< 0). It should be noted that −1 ≤ *s*_*t*−1_ ≤ 1. Parameter *β* (*β* ≥ 0) determines the sensitivity of the stimulus to the reinforcement signal (i.e., *r*_*t*−1_ − *A*).For the PDG, we updated *p*_*t*_ as follows:


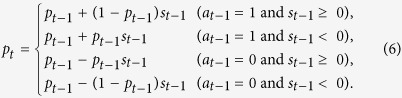


The first line in equation (6) states that, if the participant cooperates and is satisfied in round *t *− 1, the probability of cooperation increases. The factor (1 − *p*_*t*_) accounts for the fact that *p*_*t*_ cannot exceed unity. The second line states that, if the participant cooperates and is dissatisfied, the probability of cooperation decreases. The initial probability of cooperation, *p*_1_, was also estimated from the empirical data.

For the PGG, we modified equation (6) as follows:


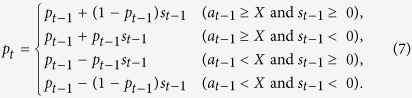


Because the action was essentially continuous in the PGG, we introduced another threshold contribution level *X* (0 ≤ *X* ≤ 1) to decide whether a realized contribution was regarded to be large (i.e., cooperative) or small (i.e., defective).

### RE model

We assumed that the total payoff to a player was nonnegative, as in our experiments. In the RE model, the probability that an action is selected is encoded by the corresponding attraction score, which is updated for all possible actions in each round.

For the PDG, the attraction score for C, denoted by *q*_1*,t*_, and that for D, denoted by *q*_0*,t*_, were updated by


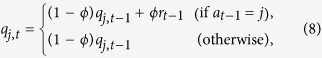


where *j* = 0, 1. We remind that *r*_*t*−1_ is the earning of the focal participant in round *t *− 1. Parameter *ϕ* determines the weight on the current payoff relative to the past ones. A large *ϕ* value corresponds to a short memory of the player. We set the initial condition to *q*_0,1_ = *q*_1,1_ = 0. A player selects C with probability


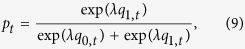


where *λ* represents the sensitivity of *p*_*t*_ to the attraction scores. If *λ* = 0, we obtain *p*_*t*_ = 1/2 regardless of the attraction scores.

For the PGG, we updated the attraction scores by


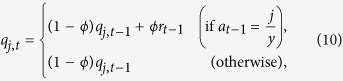


where *j* = 0, 1, 2, …, 25. Similarly to the case of the PDG, we set the initial condition to *q*_0,1_ = *q*_1,1_ = …  = *q*_25,1_  = 0. The probability that a player contributes *j* in round *t*, denoted by *p*_*j,t*_, is given by


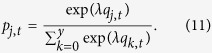


We calculated the expectation of the normalized amount of contributions in round *t* by


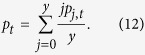


### Estimation of parameter values

Using maximum likelihood, we fitted each of the four models (i.e., CC, MCC, BM, and RE) to each of the four data sets (fixed × PDG, mixed × PDG, fixed × PGG, mixed × PGG). Each data set contained 2,000 decisions (100 participants × 20 rounds). We used the *n* = 1,900 decisions in round *t *> 1 (i.e, 100 participants × 19 rounds) as dependent variables. In the PDG, we assumed that the *i*th decision (1 ≤ *i* ≤ *n*) obeyed the Bernoulli distribution with probability of cooperation *p*_*i*_. The likelihood, denoted by *L*, is given by


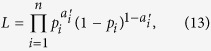


where 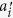 represents the *i*th action (C = 1, D = 0) realized in the experiment. In the PGG, *p*_*i*_ represents the expectation of the normalized contribution. We assumed that the realized normalized contribution, denoted by 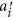 (0 ≤ 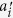 ≤ 1), obeyed the Gaussian distribution. The likelihood for the PGG is given by


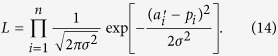


We also estimated the value of *σ*^2^. We calculated the values of parameters that maximized log *L* using optim function with L-BFGS-B method implemented in R 3.0.2.

### Model selection

We calculated the AIC defined by


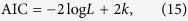


where *k* is the number of parameters in a model. We also calculated the mean squared error (MSE) given by


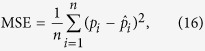


where *p*_*i*_ and 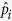 are the observed and estimated values of the probability of cooperation, respectively.

## Additional Information

**How to cite this article**: Horita, Y. *et al*. Reinforcement learning accounts for moody conditional cooperation behavior: experimental results. *Sci. Rep.* **7**, 39275; doi: 10.1038/srep39275 (2017).

**Publisher's note:** Springer Nature remains neutral with regard to jurisdictional claims in published maps and institutional affiliations.

## Supplementary Material

Supplementary Information

## Figures and Tables

**Table 1:** Main symbols.

Symbol	Meaning
*N*_c_	Number of peers in the current group that cooperated in the previous round in the PDG (=0, 1, 2, or 3).
*a*_*t*_	Action of the focal participant in round *t*. In the PDG, C and D correspond to *a*_*t*_ = 1 and *a*_*t*_ = 0, respectively. In the PGG, *a*_*t*_ (0 ≤ *a*_*t*_ ≤ 1) is the normalized contribution, i.e., the amount of contribution divided by the possible maximum amount.
*K*_*t*−1_	Normalized amount of cooperation imparted by the other three members in the group in the previous round (0 ≤ *K*_*t*−1_ ≤ 1). In the PDG, *K*_*t*−1_ is equal to the fraction of the three members that has selected C in the previous round (i.e., *N*_c_/3). In the PGG, *K*_*t*−1_ is equal to the normalized contribution in the previous round averaged over the three members.
*p*_*t*_	The propensity to cooperate in the present round. In the PDG, *p*_*t*_ is the probability of selecting C. In the PGG, *p*_*t*_ is the expected normalized contribution.

**Table 2:** Model selection.

PDG	log *L*	AIC	MSE [CI]
**Fixed treatment**			
CC	−1209.44	2422.88	0.22 [0.22, 0.23]
MCC	−1014.31	2036.63	0.18 [0.17, 0.19]
BM	−947.67	1901.34	0.16 [0.15, 0.17]
RE	−947.40	1898.81	0.16 [0.15, 0.17]
**Mixed treatment**			
CC	−1137.40	2278.79	0.20 [0.20, 0.21]
MCC	−930.35	1868.70	0.16 [0.15, 0.17]
BM	−757.76	1521.51	0.13 [0.12, 0.14]
RE	−803.37	1610.74	0.13 [0.13, 0.14]
**PGG**	**log** ***L***	**AIC**	**MSE [CI]**
**Fixed treatment**			
CC	−654.76	1315.51	0.12 [0.11, 0.12]
MCC	−352.50	715.01	0.08 [008, 0.09]
BM	−404.98	819.95	0.09 [0.08, 0.10]
RE	−369.29	744.58	0.09 [0.08, 0.09]
**Mixed treatment**			
CC	−375.37	756.75	0.09 [0.08, 0.09]
MCC	84.88	−159.76	0.05 [0.05, 0.06]
BM	113.97	−217.94	0.05 [0.05, 0.06]
RE	114.03	−222.07	0.05 [0.05, 0.06]

The values in the parentheses are 95% confidential intervals. CC: conditional cooperation. MCC: moody conditional cooperation, BM: Bush-Mosteller model of reinforcement learning. RE: Roth-Erev model of reinforcement learning.
