# Supplementary material for: Reinforcement learning accounts for moody conditional cooperation behavior: experimental results
Source: Sci Rep. 2017 Jan 10;7:39275. doi: 10.1038/srep39275 (PMC5223288; doi:10.1038/srep39275)
Supplement: Supplementary Information [file srep39275-s1.pdf]

Supporting Information for “Reinforcement learning accounts for moody conditional cooperation behavior: experimental results”

Yutaka Horita, Masanori Takezawa, Keigo Inukai, Toshimasa Kita, Naoki Masuda

Supplementary Figures
Figure S1: Decision screens presented to the participants
Figure S2: The relationship between the level of cooperation in the PDG and that in the PGG
Figure S3: The expected probability of C predicted by the reinforcement learning models in the PDG
Figure S4: The expected fraction of contributions predicted by the reinforcement learning models in the PGG

Supplementary Tables
Table S1: Maximum likelihood estimators obtained for the behavioral data in the PDG
Table S2: Maximum likelihood estimators obtained for the behavioral data in the PGG
Table S3: Results of fitting the directional learning model to the PGG data

Supplementary Methods
Values of the payoffs used in the experiments
Directional learning model

Supplementary Figures

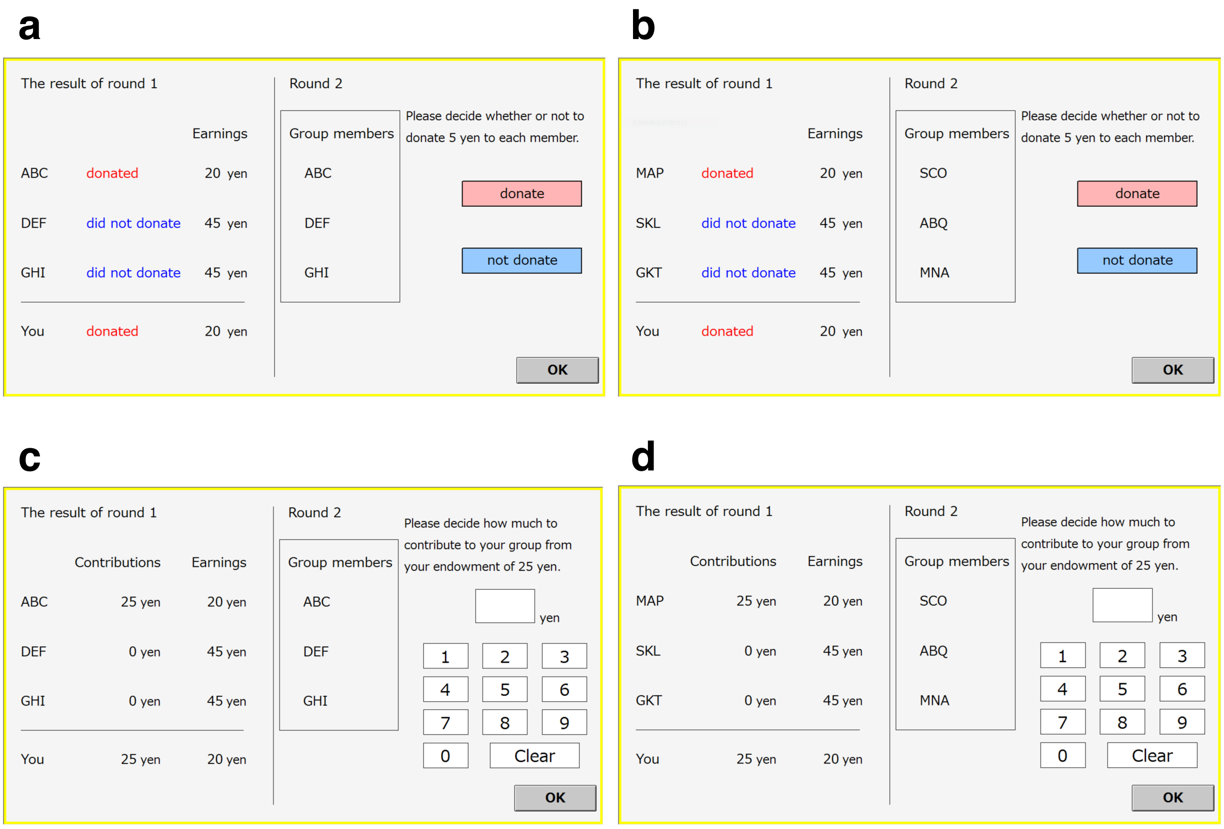

Supplementary Figure S1: Decision screens presented to the participants. (a) PDG in the fixed treatment, (b) PDG in the mixed treatment, (c) PGG in the fixed treatment, and (d) PGG in the mixed treatment. In fact, the screens were displayed in Japanese. Here we translated the text into English.

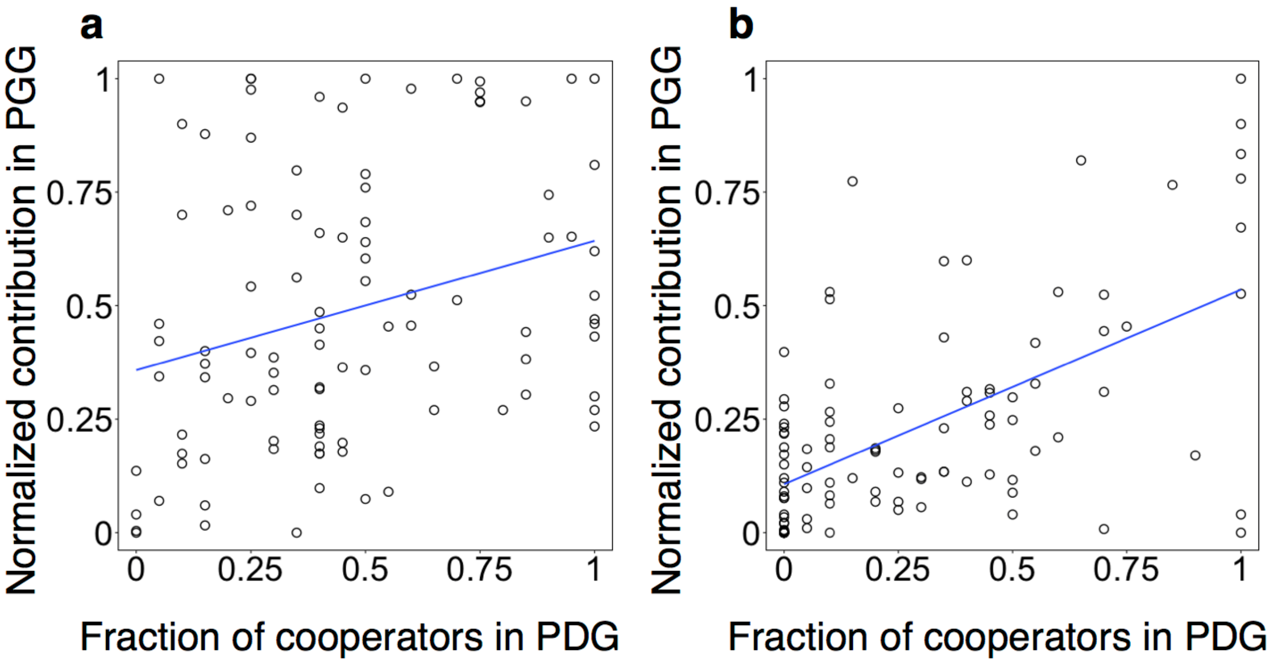

Supplementary Figure S2: The relationship between the level of cooperation in the PDG and that in the PGG. In each type of game, the level of cooperation for each participant was calculated as an average over 20 rounds. A circle represents a participant. The solid lines represent the linear regression. (a) Fixed treatment. (b) Mixed treatment.

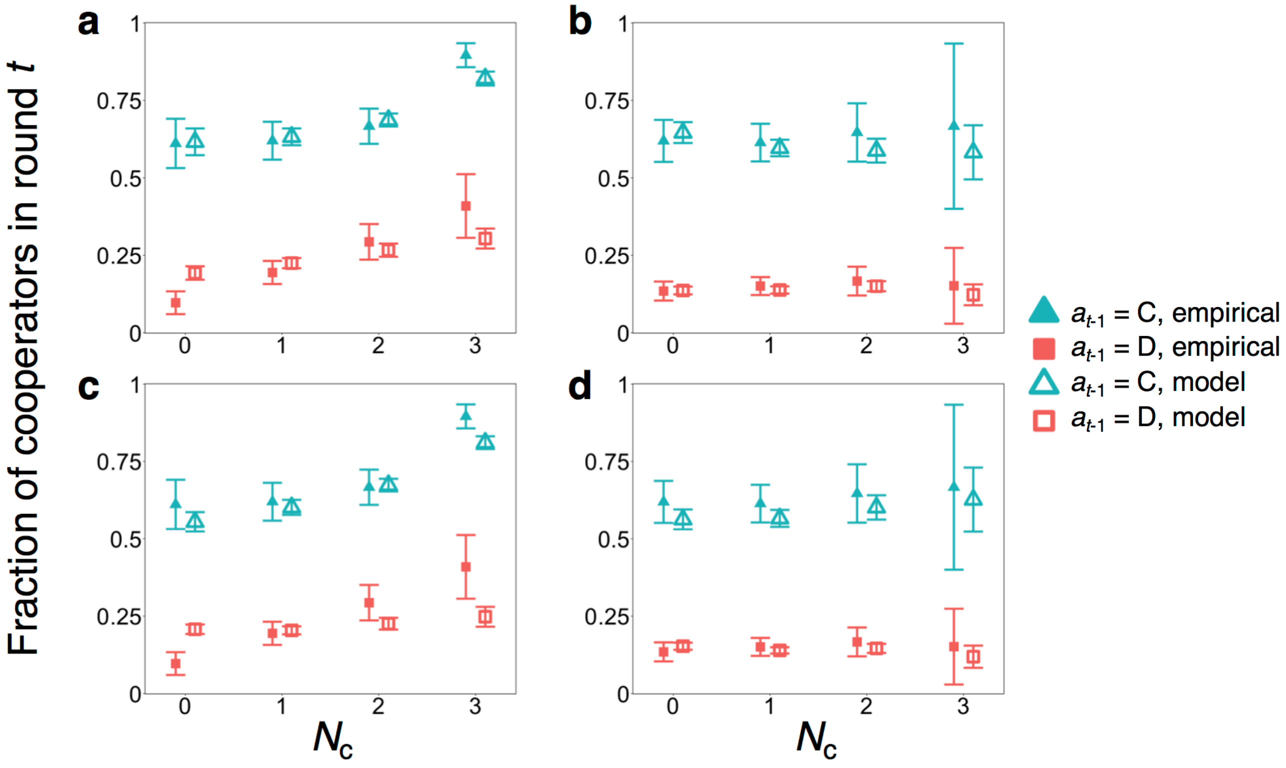

Supplementary Figure S3: The expected probability of C predicted by the reinforcement learning models in the PDG. We predicted the probability of C by running the BM or RE model with the estimated parameter values. Then, we averaged the predicted probability of cooperation over the participants and the rounds, and plotted them as a function of N_c (i.e., the number of the other group members that cooperated in the previous round). (a) BM model in the fixed treatment. (b) BM model in the mixed treatment. (c) RE model in the fixed treatment. (d) RE model in the mixed treatment. The triangles and squares represent the probability of C conditioned on a_{t-1} = C and a_{t-1} = D, respectively. Filled symbols represent the empirical data. Open symbols represent the prediction by the model. The error bars represent the 95% confidence intervals.

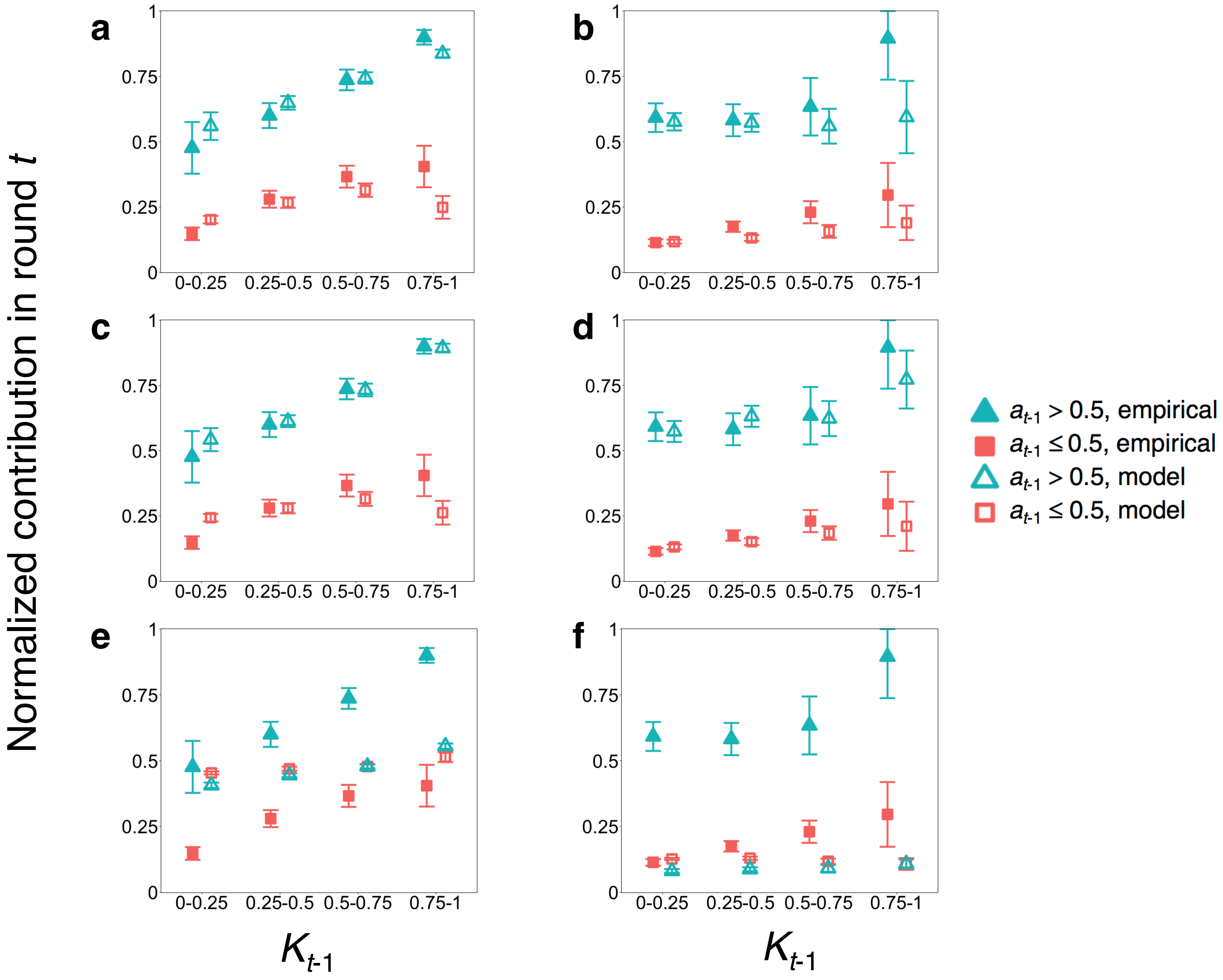

Supplementary Figure S4: The expected fraction of contributions predicted by the reinforcement learning models in the PGG. We predicted the fraction of contributions by running the BM, RE, or directional learning model with the estimated parameter values. Then, we averaged the predicted fraction of contributions over the participants and the rounds, and plotted them as a function of K_{t-1} (i.e., the average fraction of contribution by the other group members in the previous round). (a) BM model in the fixed treatment. (b) BM model in the mixed treatment. (c) RE model in the fixed treatment. (d) RE model in the mixed treatment. (e) Directional learning model in the fixed treatment. (f) Directional learning model in the mixed treatment. The triangles and squares represent the fraction of contributions among high ($a_{t\text{-}1} > 0.5$) and low ($a_{t\text{-}1} \leq 0.5$) contributors in the previous round, respectively. Filled symbols represent the fraction of contributions obtained from the empirical data. Open symbols represent the expected fraction of contribution predicted by the model. The error bars represent the 95% confidence intervals.

Supplementary Table S1: Maximum likelihood estimators obtained for the behavioral data in the PDG. CC: conditional cooperation. MCC: moody conditional cooperation. BM: Bush-Mosteller model. RE: Roth-Erev model. For the CC and the MCC models, the 95% confidence interval for each parameter is shown in parentheses.

		Fixed [CI]	Mixed [CI]
CC	α_1	2.01 [1.71, 2.31]	0.16 [–0.21, 0.54]
	α_2	–1.19 [–1.36, –1.01]	– 0.96 [–1.11, –0.81]
MCC	α_1	1.81 [1.30, 2.32]	0.32 [–0.25, 0.88]
	α_2	–2.09 [–2.38, –1.80]	–1.85 [–2.08, –1.62]
	α_3	2.22 [1.83, 2.61]	2.30 [1.96, 2.65]
	α_4	–0.37 [–1.04, 0.30]	–0.16 [–1.03, 0.71]
BM	β	6.01×10^{-3}	3.27×10^{-3}
	A	–1.60	–40.47
	p_1	0.57	0.32
RE	ϕ	0.24	0.19
	λ	0.07	0.10

Supplementary Table S2: Maximum likelihood estimators obtained for the behavioral data in the PGG. CC: conditional cooperation. MCC: moody conditional cooperation. BM: Bush-Mosteller model. RE: Roth-Erev model. For the CC and the MCC models, the 95% confidence interval of each parameter is shown in parentheses.

		Fixed [CI]	Mixed [CI]
CC	α_1	3.25 [2.95, 3.54]	1.81 [1.44, 2.17]
	α_2	–1.69 [–1.86, –1.53]	–1.69 [–1.82, –1.55]
MCC	α_1	1.61 [1.15, 2.07]	1.43 [0.89, 1.96]
	α_2	–2.13 [–2.39, –1.88]	–2.52 [–2.72, –2.33]
	α_3	2.09 [1.68, 2.49]	3.24 [2.89, 3.60]
	α_4	0.75 [0.03, 1.46]	–0.38 [–1.42, 0.66]
BM	β	5.55×10^{-3}	3.09×10^{-3}
	A	5.07×10^{-4}	–23.96
	X	0.50	0.50
	p_1	0.56	0.30
RE	ϕ	0.28	0.24
	λ	0.24	0.43

Supplementary Table S3: Results of fitting the directional learning model to the PGG data. The 95% confidence interval is shown in parentheses.

		Fixed	Mixed
Parameter values	β	2.95×10^{-3}	1.14×10^{-3}
	A	24.91	15.00
	p_1	0.43	0.03
log L		−912.43	−624.79
AIC		1832.86	1257.59
MSE [CI]		0.16 [0.16, 0.17]	0.12 [0.11, 0.13]

Supplementary Methods

Values of the payoffs used in the experiments

We determined the values of b, c, and m to make the payoff when a player chose C or D in the PDG be identical to that when the player maximally cooperated or maximally defected in the PGG, respectively. Denote by N the number of participants in the group. Denote by K the fraction of players who select C in the case of the PDG and the normalized contribution averaged over the group members in the case of the PGG. Denote by $\tilde{a}_i$ the action (C = 1, D = 0) by the ith other member in the group ($1 < i \leq N - 1$) in the case of the PDG and the normalized contribution by the ith other member in the group ($0 \leq \tilde{a}_i \leq 1$) in the case of the PGG. Then, we obtain $K = \sum_{i=1}^{N-1} \tilde{a}_i/(N-1)$.

First, consider the case in which a player maximally cooperates in both the PDG and the PGG. By equating the payoff value given by equation (1) with $a_t = 1$ and that given by equation (2) with $a_t = 1$, we obtain

$$y - (N-1)c + bK(N-1) = \frac{m}{N}y[K(N-1)+1]. \tag{S1}$$

Second, consider the case in which a player maximally defects in both the PDG and the PGG. By equating equation (1) with $a_t = 0$ and equation (2) with $a_t = 0$, we obtain

$$y + bK(N-1) = y + \frac{m}{N}yK(N-1). \tag{S2}$$

By combining equations (S1) and (S2), we obtain

$$b = \frac{m}{N}y \tag{S3}$$

and

$$(N-1)c = y - b, \tag{S4}$$

which yield

$$\frac{b}{c} = \frac{(N-1)m}{N-m}. \tag{S5}$$

Substitution of $N = 4$ and $b/c = 2$ in equation (S5) yields $m = 1.6$.

Directional learning model

In the directional learning model, the stimulus, s_t, was defined by equation (5), and equation (7) was modified as follows:

$$p_t = \begin{cases} p_{t-1} + (1-p_{t-1})s_{t-1} & (a_{t-1} \geq a_{t-2} \text{ and } s_{t-1} \geq 0), \\ p_{t-1} + p_{t-1}s_{t-1} & (a_{t-1} \geq a_{t-2} \text{ and } s_{t-1} < 0), \\ p_{t-1} - p_{t-1}s_{t-1} & (a_{t-1} < a_{t-2} \text{ and } s_{t-1} \geq 0), \\ p_{t-1} - (1-p_{t-1})s_{t-1} & (a_{t-1} < a_{t-2} \text{ and } s_{t-1} < 0). \end{cases} \tag{S6}$$

The first line of equation (S6), for example, states that, if a participant has increased the contribution in the last round (i.e., $a_{t\text{-}1} \geq a_{t\text{-}2}$) and been satisfied ($s_{t\text{-}1} \geq 0$), then the participant increases the amount of contribution (i.e., p_t).
